# Performance Enhancement of a Web-Based Picture Archiving and Communication System Using Commercial Off-the-Shelf Server Clusters

**DOI:** 10.1155/2014/657417

**Published:** 2014-02-20

**Authors:** Yan-Lin Liu, Cheng-Ting Shih, Yuan-Jen Chang, Shu-Jun Chang, Jay Wu

**Affiliations:** ^1^Institute of Nuclear Engineering and Science, National Tsing Hua University, Hsinchu 30013, Taiwan; ^2^Department of Biomedical Engineering and Environmental Sciences, National Tsing Hua University, Hsinchu 30013, Taiwan; ^3^Department of Management Information Systems, Central Taiwan University of Science and Technology, Taichung 40202, Taiwan; ^4^Health Physics Division, Institute of Nuclear Energy Research, Atomic Energy Council, Taoyuan 32500, Taiwan; ^5^Department of Biomedical Imaging and Radiological Science, China Medical University, No. 91 Hsueh-Shih Road, Taichung 40202, Taiwan

## Abstract

The rapid development of picture archiving and communication systems (PACSs) thoroughly changes the way of medical informatics communication and management. However, as the scale of a hospital's operations increases, the large amount of digital images transferred in the network inevitably decreases system efficiency. In this study, a server cluster consisting of two server nodes was constructed. Network load balancing (NLB), distributed file system (DFS), and structured query language (SQL) duplication services were installed. A total of 1 to 16 workstations were used to transfer computed radiography (CR), computed tomography (CT), and magnetic resonance (MR) images simultaneously to simulate the clinical situation. The average transmission rate (ATR) was analyzed between the cluster and noncluster servers. In the download scenario, the ATRs of CR, CT, and MR images increased by 44.3%, 56.6%, and 100.9%, respectively, when using the server cluster, whereas the ATRs increased by 23.0%, 39.2%, and 24.9% in the upload scenario. In the mix scenario, the transmission performance increased by 45.2% when using eight computer units. The fault tolerance mechanisms of the server cluster maintained the system availability and image integrity. The server cluster can improve the transmission efficiency while maintaining high reliability and continuous availability in a healthcare environment.

## 1. Introduction

In the last decade, picture archiving and communication systems (PACSs) have been proven to be an effective platform for increasing productivity and patient satisfaction in healthcare facilities [1–4]. In all types of PACS architecture, the Web-based model enables hospital- and campus-wide image distribution and management [5–8]. It has been accepted as a primary option for large-scale PACSs. However, central archiving and on-demand viewing of images cause a heavy burden on the PACS server and a burst of network traffic during peak hours [9]. The image delivery time is substantially prolonged and may not be accepted by clinicians. Therefore, increasing system efficiency as well as maintaining reliability is necessary for a robust Web-based PACS.

A Web-based PACS is time sensitive. Prompt delivery of medical images is one of the most critical tasks in maintaining radiology workflow. Two kinds of methods exist to increase the image distribution speed: the use of a faster network connection and the deployment of a high-performance server. For the network connection, gigabit Ethernet or higher is essential for image transfer from the PACS archiver to display workstations [10]. Furthermore, a network with a minimum speed of 100 Mbit/s should be connected between the imaging modalities and the PACS server [11, 12]. For the server performance, the time-to-display and time-to-upload of various server hardware configurations were investigated [9, 11]. Increasing the amount of RAM and the number of CPUs can lead to a substantial decrease in transmission time.

In addition to improving the system efficiency, maintaining the reliability and validity of PACSs is also critical. The PACS server is a single point of failure (SPOF); any interruption of its services could jeopardize the data integrity and hamper daily clinical operations. Therefore, fault tolerance measurements should be taken to maximize the system uptime for end users. A simple method is to use spares, which can achieve an availability rate of 99% [13]. An alternative approach is to deploy cluster servers [14, 15], which can reach availability of 99.99%. A triple modular redundant (TMR) architecture with three Solaris Unix servers has been used to construct a robust PACS with high operational reliability [16]. A continuous availability of 99.999% was achieved in a variety of clinical situations. Although adding redundant equipment is straightforward, it is expensive and does not improve the image transmission speed. Other studies have demonstrated the backup and recovery of clinical images by using a data grid architecture [17] and an Application Service Provider (ASP) model [18].

Considering the cost and effectiveness, commercial off-the-shelf (COTS) hardware was used in this study to build a server cluster that features an active/active configuration, running the network load balancing (NLB), distributed file system (DFS), and structured query language (SQL) server replication services. We compared the average transmission rate between various configurations of PACS servers with a variety of scenarios, including image upload, download, and server failure, as a benchmark of efficiency improvement. The purpose of this study was to evaluate the feasibility of using the COTS server cluster as a sophisticated PACS archiving and controller server to increase system efficiency and reliability in a healthcare environment.

## 2. Materials and Methods

### 2.1. Server Configuration

The hardware configuration of the COTS PACS server consisted of an AMD Athlon 64 X2 4200+ dual-core processor (2.2 GHz) and 8 GB of DDRII RAM (533 MHz). Two network interface cards were installed; one (Broadcom 5755 Gigabit Ethernet) handled the interserver communication and the other (IntelPRO/100 Management Adapter 82559) handled the network traffic to the Ethernet backbone. Four 250 GB hard drives, creating a total usable storage of 750 GB RAID 5 array, were attached to the server as a short-term storage device. The server was running on Windows Server 2008 R2 with Service Pack 1 installed. Conquest software version 1416rc2 was implemented as the image Web server (IWS). The functions of Conquest include image indexing, image archiving, image format conversion, DICOM network access, DICOM image filtering and web viewing, and image compression. The Microsoft SQL Server 2008 was also employed as required by the IWS to index and store details of the patient information, examination study, series number, image modality, and incoming images as the database for image query.

### 2.2. Client Configuration

Personal computers with an Athlon X2 4200+ CPU and 1 GB of RAM were deployed as client workstations. A Broadcom 5755 Gigabit Ethernet card and a 160 GB hard drive were installed. Microsoft Windows XP Professional was installed as the operating system, and Internet Explorer 6.0 was used as the standard Web browser to access the IWS via DICOM Query and Retrieve protocol. When the client computer logged into the IWS for the first time, an ActiveX component was loaded to enable the query/retrieve, patient management, and image viewing and modification functions.

### 2.3. Cluster and Noncluster Modes of Servers

**Figure 1:**
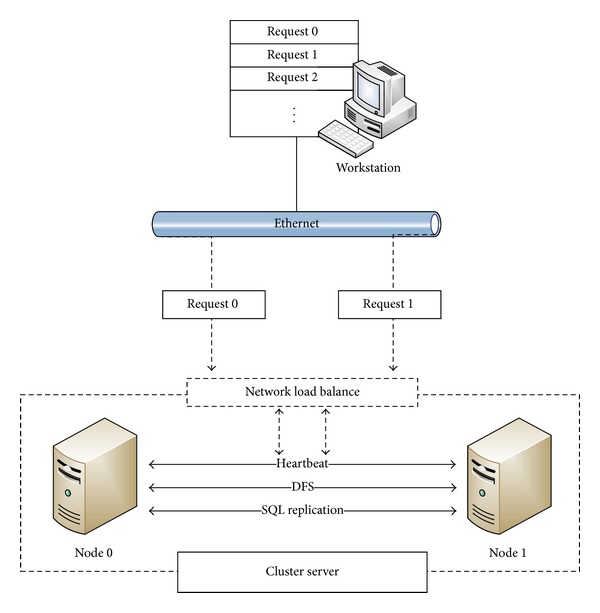
System architecture of the cluster server with two computer nodes running the NLB, DFS, and SQL services provided by Microsoft Windows Server 2008 in the PACS environment. Each node also runs the Conquest software as the IWS and servers as the archive server for short-term storage.

Two types of server architecture were constructed: the noncluster and cluster modes. For the noncluster mode, one server computer was used to fulfill the requests of image upload and download from modalities and client workstations, whereas for the cluster mode, two identical server computer nodes were grouped to form an active/active server cluster as the PACS archive server.  Figure 1 illustrates the cluster architecture in our study.

In addition to installing the IWS in the server computers, the NLB, DFS, and SQL server replication services [19, 20] provided by Windows Server 2008 were installed and activated for the cluster mode. For the NLB service, the unicast mode of operation was selected, allowing periodic interhost communication through heartbeat messages. For the DFS service, the server nodes were set as a replication group participating in synchronization of a DICOM folder which stores the incoming images. When an image is transferred from a workstation to one of the server node, it is replicated across intranet between the members of the replication group. The SQL server replication service was activated for copying, distributing, and synchronizing database objects within the server cluster. The merge replication was applied in the cluster environment to enable multiple subscribers to update data simultaneously.

### 2.4. Performance Measurement

During the data transmission, Windows Performance Monitor was used on the client side and server side to record the network traffic and the CPU usage. The average transmission rate (ATR) was measured and calculated as a performance index as follows:

(1) $$\begin{array}{c} \text{ATR}=\frac{n\times s}{\sum_{i=1}^{n}t_{i}}, \end{array}$$

where *t*_*i*_ is the transmission time of the *i*th client computer, *s* is the total size of image transmission per client, and *n* is the number of clients performing image upload or download tasks.

### 2.5. Transmission Scenarios

We built the cluster server environment described in Section 2.3 in our hospital and distributed the client workstations over the radiological department to simulate upload, download, mix, and disaster scenarios. These scenarios are described as follows.

#### 2.5.1. Upload Scenario

The image upload was performed using 1 to 16 client computers to transfer images to the PACS archive server. Computed radiography (CR), computed tomography (CT), and magnetic resonance (MR) images with a total file size of 300 MB each were transferred (Table 1). The ATR was then analyzed for both cluster and noncluster server configurations. The client computers were preinstalled with a homemade application to support the DICOM objects of CR, CT, and MR and the storage service class user (SCU) and service class provider (SCP) [21].

#### 2.5.2. Download Scenario

Various numbers of client computers, from 1 to 16, were used to download images from the PACS server. The client computer was first logged into the IWS. It then queried the PACS server and retrieved three series of DICOM images, which were exactly the same as those used in the upload scenario. Finally, the received image packets were restored and displayed on a monitor consecutively. The ATRs were measured and compared between the cluster and noncluster configurations.

#### 2.5.3. Mix and Disaster Scenarios

In the mix scenario, two client computers were grouped as one unit in which one computer performed the image download procedure and the other performed the image upload procedure. A total file size of 160 MB, consisting of CR, CT, and MR images, was transferred (Table 2). Various numbers of units, from 1 to 8, were used to execute their tasks. The purpose of the mix scenario was to simulate a real healthcare environment. Additionally, the disaster scenario was simulated with 8 units executing the mix scenario. The connection of one server node was removed after 10, 20, and 40 s to simulate a failover situation. The transactional integrity of the images was analyzed as well as the ATR. All measurements were repeated in triplicate, and the mean and standard deviations were estimated.

### 2.6. Auxiliary Software

In addition to the aforementioned software and services, time synchronization and automatic operation of the client computers were required to reduce human error. NTPClock version 2.1 which supports the Network Time Protocol (NTP) was installed on the client and server computers. This software sends periodic requests to the server located in the National Standard Time and Frequency Laboratory, Taiwan, to adjust the clock in the operating system, achieving an accuracy of 30 ms. AutoMouse version 1.3 [22] was installed to automatically control the client computers. The mouse movements, mouse clicks, and keyboard strikes for each scenario were recorded in advance. Therefore, the client computers can replay the exact commands and procedures at the appropriate times.

## 3. Results

### 3.1. Download Scenario

**Figure 2:**
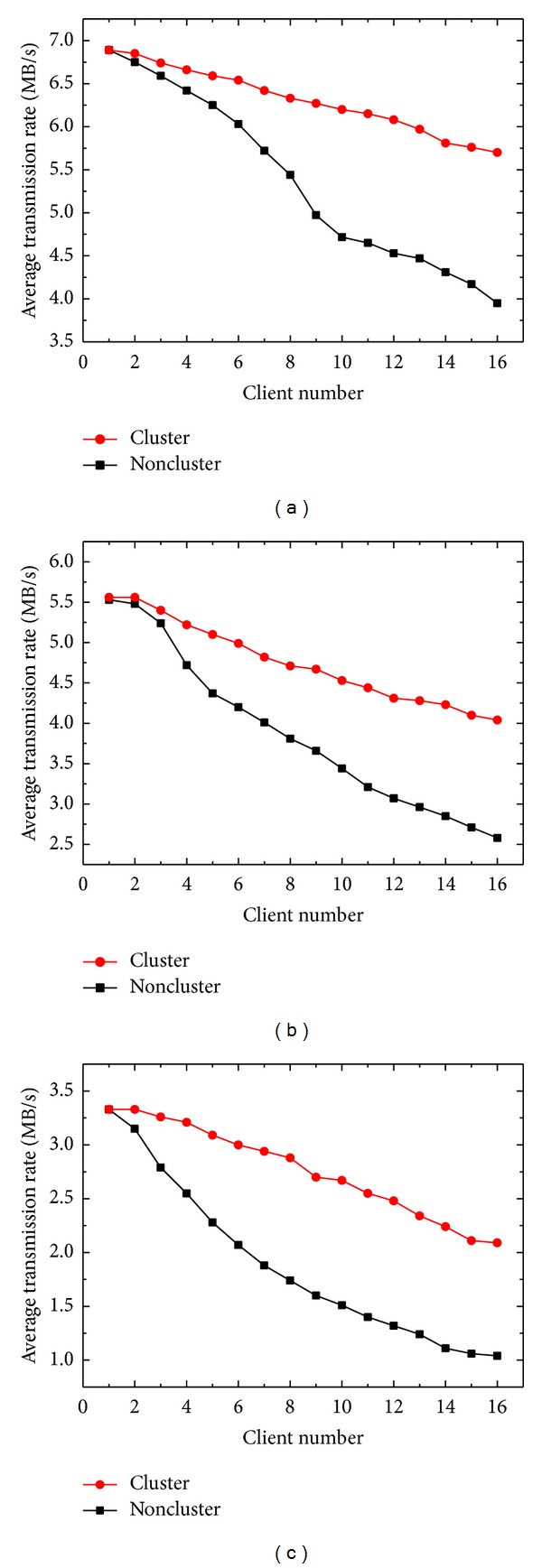
ATRs of the cluster and noncluster modes as a function of client number for downloading (a) CR, (b) CT, and (c) MR images.

Figure 2 shows the ATRs of the cluster and noncluster modes as a function of client number for downloading CR, CT, and MR images. A downward trend of ATR was observed for both cluster modes with an increase in the client number. When the client number was less than two, the difference between the two modes was less than 6%. As the client number increased, the cluster mode consistently exhibited stronger performance than the noncluster mode. The maximum differences were 44.3%, 56.6%, and 100.9% for CR, CT, and MR images, respectively, when 16 clients downloaded images simultaneously. The performance improvement is mainly because the NLB service can successfully divert download jobs to different cluster nodes, which reduces the loading of the server.

### 3.2. Upload Scenario

**Figure 3:**
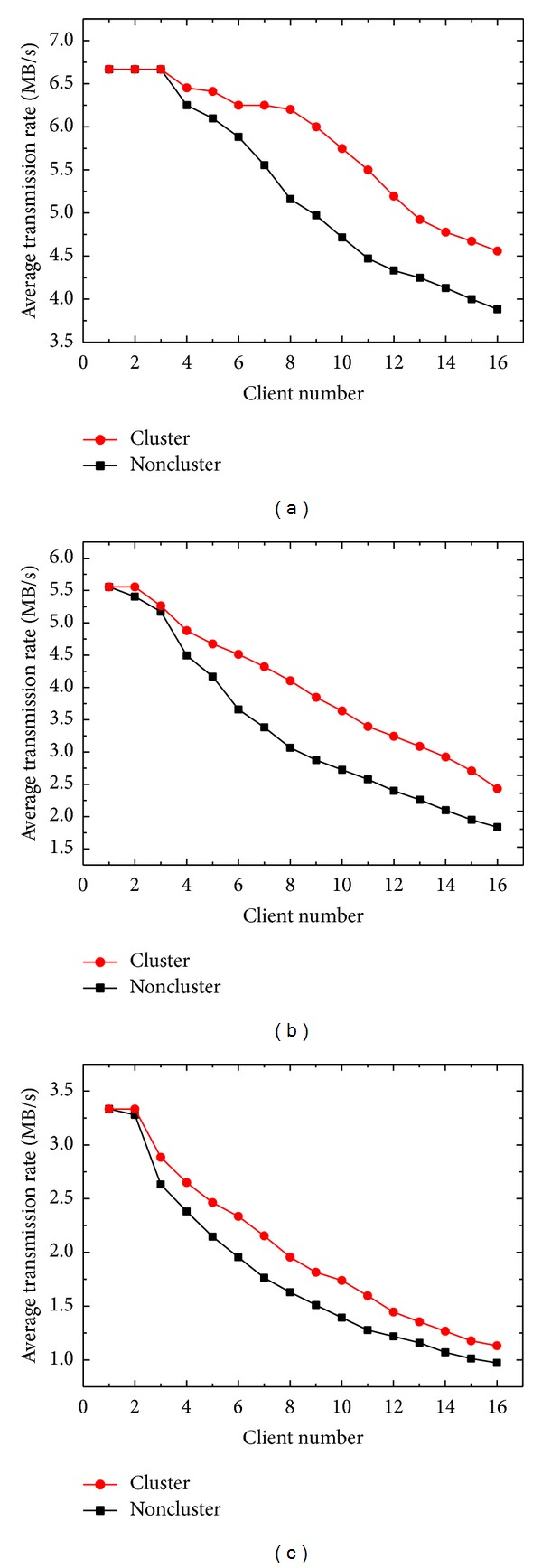
ATRs of the cluster and noncluster modes as a function of client number for uploading (a) CR, (b) CT, and (c) MR images.

Figure 3 shows the ATRs of the cluster and noncluster modes when various numbers of client computers uploaded CR, CT, and MR images. Initially, the curves of the cluster and noncluster modes were comparable. As the client number increased, the ATRs of the noncluster mode decreased more rapidly than those of the cluster mode, and the differences between two curves became obvious. The maximum differences reached 23.0%, 39.2%, and 24.9% for CR, CT, and MR images at 11, 14, and 11 clients, respectively. As the client number further increased to 16, the differences in ATRs between modes reduced to 17.3%, 32.4%, and 16.4%. Additionally, the cluster configuration was less effective in the upload scenario than in the download scenario. The main reason is that the images uploaded to the server are required to be synchronized between nodes by the DFS and SQL replication services, which creates additional burden on the server CPU.

**Figure 4:**
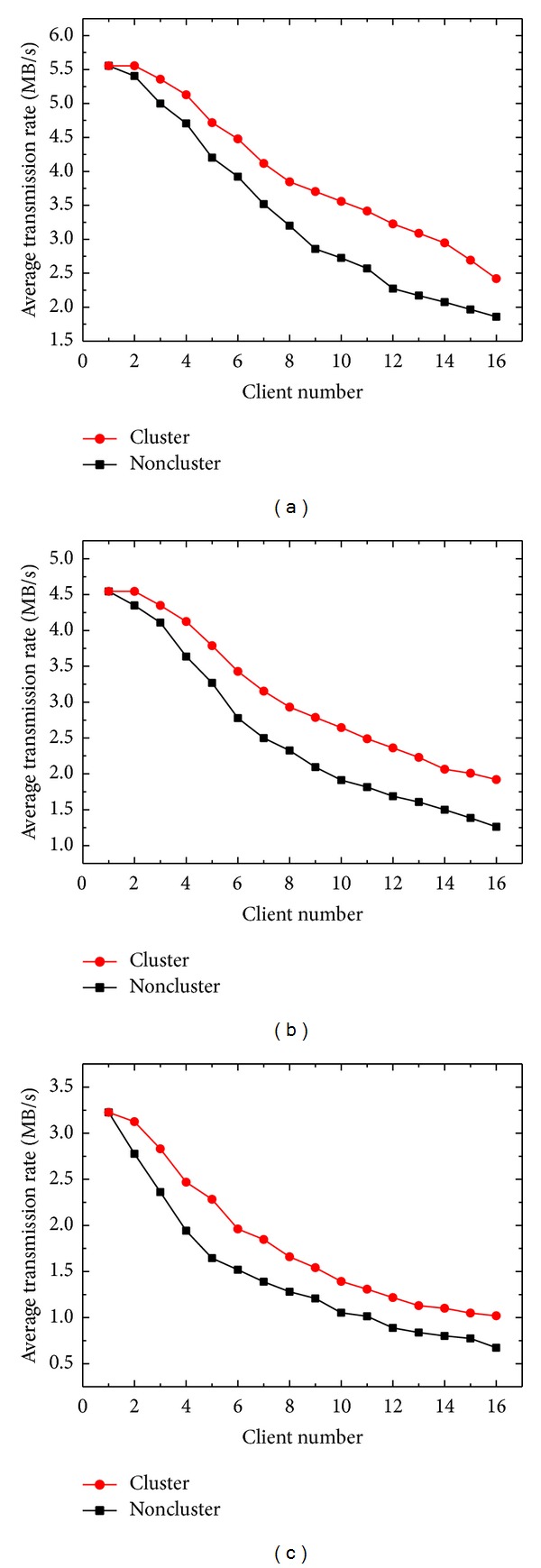
ATRs of the cluster and noncluster modes as a function of client number for uploading (a) CR, (b) CT, and (c) MR images with image compression.

Figure 4 shows the ATRs of uploading CR, CT, and MR images when image compression was performed on the server side. Compared to the noncompression condition in Figure 3, the ATRs for both modes declined markedly. In the noncluster mode, ATRs decreased to 1.86, 1.26, and 0.67 MB/s for the CR, CT, and MR images, respectively, when the client number reached 16. This is primarily because of the extra CPU loading required for the data compression routine. In this situation, the cluster mode can still improve the performance by 42.2%, 45.1%, and 49.9% for CR, CT, and MR images, respectively. The improvement results were superior to those without image compression.

### 3.3. Mix Scenario

**Figure 5:**
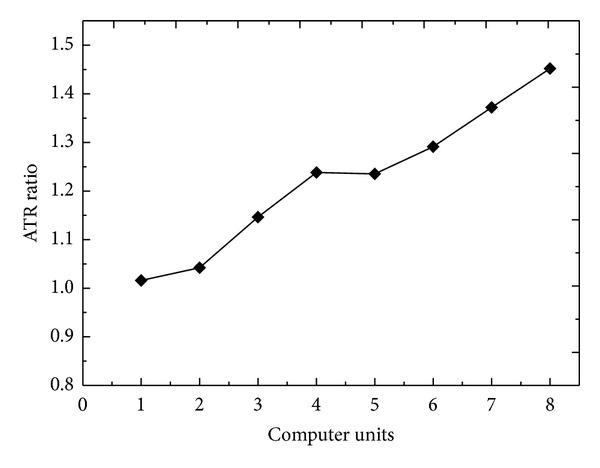
ATR ratios between the cluster and noncluster modes for the mix scenario. The ATR ratio increased in conjunction with the computer units.

Figure 5 illustrates the ATR ratios between the cluster and noncluster modes for the mix scenario. A computer unit consists of two computers; one performs image download and the other simultaneously performs image upload. The curve rose slowly at the initial stage. When the unit number increased to eight, the ATR ratio increased to 1.45 and the difference in ATR between two modes was 1.22 MB/s. Compared with the download and upload scenarios, the mix scenario more realistically represents the actual transmission conditions in healthcare facilities. The cluster mode can improve the performance by approximately 23.8%, 29.1%, and 45.2% at 4, 6, and 8 computer units.

### 3.4. Disaster Scenario

**Figure 6:**
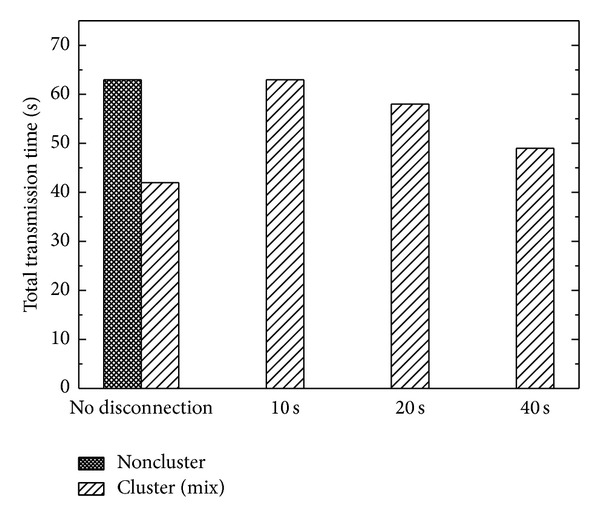
Total transmission times for the disaster scenario. The connection of one server node was removed after 10, 20, and 40 s to simulate a failover situation.

Figure 6 shows the total transmission time for the disaster scenario. The transmission conditions were the same as those in the mix scenario with eight computer units uploading and downloading images simultaneously. The cluster and noncluster modes required 42 and 63 s, respectively, to transfer all the images. Once the Ethernet connection of one node in the cluster was interrupted after 10, 20, and 40 s, the transmission corresponding to the interrupted node was stopped and the remaining workload was automatically diverted to the healthy node. Although the transmission times were prolonged to 63, 58, and 49 s accordingly, none of the images were missing during the server down time. This indicates that the cluster server can maintain the continuous availability and data integrity even if one of the nodes fails.

## 4. Discussions

Most clinical PACS servers use Unix-based architecture because of the reliability in hardware and software infrastructure. However, replacement parts for Unix servers are expensive and must be purchased from PACS vendors, causing inconvenience in maintaining the host machine. Using COTS hardware and a Windows server system as a PACS server is relatively cheap and easy to maintain. However, the efficiency, capacity, reliability, and scalability of this type of PACS are frequently questioned. Wendt et al. [14] constructed a PACS server using COTS hardware in an online clinical environment. Their system can minimize the PACS downtime at an event of failover. However, the reliability and capacity of the PACS system are not evaluated. In this study, we constructed a COTS server cluster which can improve the upload and download efficiency while maintaining reliability and availability. Additionally, actual transmission rates were collected in the clinical PACS environment.

Image download is a time-demanding process. Any additional waiting time could be unacceptable for clinicians, particularly when the transmission rate is lower than 500 kB/s [10]. Previously, to solve the problem of slow access to medical images during peak hours, multiple independent picture archiving servers and IWS were used to spread the workload [23]. In this aspect, the server cluster architecture proposed in this study can substantially shorten the transmission time by using the NLB service to distribute the CPU loading. The parallel processing elevates the efficiency of downloading MR images by 100.9%. In the clinical situation, image download and upload often occur simultaneously. The proposed server cluster still has the ability to enhance the transmission speed by approximately 45.2% for MR images. These results suggest that the COTS server cluster is a viable option for Web-based PACSs.

The performance enhancement of the server cluster is related to the types of task and the number of concurrent client workstations. When the number of uploading clients increases, the transmission speed improves only slightly. This is because the files and the databases must be synchronized through the DFS and the SQL replication services, thereby causing a heavy burden on server nodes. Subsequently, the benefit of using the server cluster PACS is gradually compromised. Zhang et al. [24] measured the DFS performance in the Linux system with various concurrent users reading and writing files. The results also showed that significant CPU loads were observed when the number of users increased. Image types and file sizes have a considerable impact on upload capacity. The ATRs for uploading CT and MR images are lower than those for CR images, resulting in reduced upload capacity for these sectional images. Bergh et al. [25] evaluated the performance of various PACS servers on upload capacity. Their results also indicated that the upload efficiency for CR images was higher than that of CT and MR images.

Small- and medium-scaled hospitals require a minimum 50 GB/day upload capacity, whereas a minimum 100 GB/day capacity is required by large healthcare centers [25]. Our results for the upload scenario show that a minimum 357 GB/day upload capacity can be achieved by using the proposed server cluster. Therefore, the server cluster architecture can be applied to modern hospitals to satisfy the needs of multislice transmission, such as images produced by multidetector computed tomography (MDCT). Additionally, image compression is typically performed to avoid wasting storage space. However, compression causes the upload capacity to decrease because of extra loading to the server CPU. The server cluster architecture can effectively divert the workload to both nodes which substantially improves the upload performance.

Theoretically, increasing the nodes of the server cluster could improve transmission efficiency. However, to maintain image availability and reliability, the uploaded images have to be compared and duplicated to each node, which decreases the system performance. Therefore, healthcare facilities have to optimize the server configurations by considering their scales to achieve the optimal cost-effectiveness ratio. Additionally, the peer-to-peer (P2P) protocols [26] can be implemented to replace the traditional database storage protocols. The DFS namespace technique [27] can be used to group the shared folders located on different nodes, so that the onerous replication task can be avoided.

In the proposed server cluster, all nodes are active. Every participating node requires a monitoring script, which repeatedly checks the system status and calls the NLB utility to add or remove itself from the cluster as required. In a failover event, the NLB service automatically detects the errors and redirects the data flow. The remaining active node performs the additional processing operations. Therefore, no interruption of PACS services occurs, and users of the client computers are unaware of the failover. When we reduce the periodic checking interval of heartbeat messages, the communication between server nodes increases, resulting in minimization of the failover time. However, the NLB service requires CPU and network resources to check the incoming packets and make a proper load-balancing decision. If the checking interval is too short, the message packets could occupy all system resources, causing a decrease of PACS performance. After optimization, the periodic checking time was set as five seconds. The data can be redirected to the healthy node within ten seconds.

In the future, a multinode cluster server consisting of multiple active nodes, a primary passive node, and an alternative passive node can be constructed. The primary passive node is used when one of the active nodes fails or needs to be rolling-upgraded. The alternate passive node is used only if a failover event occurs and the primary passive node is unavailable. This design can maintain a minimum cost by using COTS hardware and maximize the efficiency, reliability, and availability of the PACS.

## 5. Conclusion

The PACS server is a single point of failure; any failover could jeopardize patient care and hospital operations. Using the proposed COTS server cluster as a Web-based PACS enhances the image download and upload efficiency and guarantees the continuous availability in a variety of medical image archiving and retrieval scenarios. This study proposed actual transmission rate of the COTS server in a clinical PACS environment, which can be used as reference for further constructing an efficient, scalable, and reliable active/active COTS server cluster for Web-based PACSs.

## Figures and Tables

**Table 1:** Three types of images were transferred in the upload and download scenarios.

Image type	Matrix size	Image size (MB)	Number of images	Total size (MB)
CR	1760 × 2140	7.19	41	294.8
CT	512 × 512	0.52	577	300.0
MR	256 × 256	0.13	2,308	300.0

**Table 2:** Three types of images were transferred in the mix and disaster scenarios.

Image type	Matrix size	Image size (MB)	Number of images	Total size (MB)
CR	1760 × 2140	7.19	7	50.3
CT	512 × 512	0.52	102	53.0
MR	256 × 256	0.13	410	53.3
